# Paradoxical delay of senescence upon depletion of BRCA2 in telomerase‐deficient worms

**DOI:** 10.1002/2211-5463.12109

**Published:** 2016-09-07

**Authors:** Mi‐Sun Kwon, Jaewon Min, Hee‐Yeon Jeon, Kwangwoo Hwang, Chuna Kim, Junho Lee, Je‐Gun Joung, Woong‐Yang Park, Hyunsook Lee

**Affiliations:** ^1^ Department of Biological Sciences & Institute of Molecular Biology and Genetics (IMBG)Seoul National UniversityGwanak‐GuSeoulKorea; ^2^ Samsung Genome InstituteSamsung Medical CenterSeoulKorea

**Keywords:** breast cancer susceptibility gene 2, *C. elegans*, homologous recombination, senescence, telomere length, *trt‐1*

## Abstract

BRCA2 is a multifunctional tumor suppressor involved in homologous recombination (HR), mitotic checkpoint regulation, and telomere homeostasis. Absence of Brca2 in mice results in progressive shortening of telomeres and senescence, yet cells are prone to neoplastic transformation with elongated telomeres, suggesting that BRCA2 has positive and negative effects on telomere length regulation along the path to tumorigenesis. Using *Caenorhabditis elegans* as a model, we show here that depletion of BRC‐2, an ortholog of BRCA2, paradoxically delays senescence in telomerase‐deficient mutant worms. Telomerase‐deficient worms (*trt‐1*) exhibit early replication senescence due to short telomeres. It should be noted that worms mutated in *brc‐2* are not viable as well due to massive genotoxic insults. However, when BRC‐2 is depleted by RNA interference in *trt‐1* mutant worms, the number of generations is unexpectedly increased with telomere length maintained, compared to telomerase mutants. Interestingly, depletion of other HR genes such as *rad‐51* and *rad‐54* exhibited similar effects. In worms doubly deficient of telomerase and *brc‐2*,* rad‐51*, or *rad‐54*, extra telomeric C‐circles were generated, suggesting that abrogation of HR induces an alteration in telomere environment favorable to illegitimate telomere maintenance when telomerase is absent. Collectively, absence of BRC‐2 in telomerase‐deficient background first leads to telomere shortening, followed by an induction of an as‐yet‐unknown telomere maintenance pathway, resulting in delay of senescence. The results have implications in the understanding of dysfunctional BRCA2‐associated tumorigenesis.

**Abbreviations**
ALT: alternative lengthening of telomeres
BRCA2: breast cancer susceptibility gene 2
HR: homologous recombination
RNAi: RNA interference
SSA: single‐strand annealing

The linearity of eukaryotic chromosomes poses many problems, including replication and the protection of chromosome ends against nucleolytic attacks. During neoplastic transformation, telomerase is reactivated to overcome the end replication problem. This allows cancer cells to replicate their DNA indefinitely avoiding telomere shortening. About 10% of cancer cells, however, do not possess telomerase activity. Instead, they utilize DNA recombination to elongate telomeres and are thus classified as ALT cancers 1. The precise mechanism of how ALT‐type tumorigenesis is induced, and how it is suppressed in normal cells remains unresolved.

BRCA2 is a multifunctional tumor suppressor with crucial activities in maintaining genome integrity throughout the cell cycle 2. In S/G2 phase, BRCA2 is critically required for double‐strand break (DSB) repair, homologous recombination (HR), by regulating Rad51 filament formation 3, 4. In HR, BRCA2 is responsible for proper loading of Rad51 to the damaged sites 5. Subsequently, Rad51 filament invades into the undamaged homologous strand, followed by D‐loop formation and DNA synthesis in an error‐free manner.

BRCA2 is also involved in telomere replication homeostasis as it loads Rad51 for efficient telomere replication and telomere capping 6. In addition, we previously showed that BRCA2 inhibits MRE11‐mediated resection of stalled replication forks at the lagging strand telomere synthesis 7. Therefore, loss of BRCA2 results in progressive shortening of telomeres and growth arrest 6, 7. As the shortening of telomeres triggered by BRCA2 deficiency is independent from telomerase activity and is incompatible with the fact that dysfunctional BRCA2 leads to tumorigenesis, we asked whether BRCA2 depletion has any possibility of inducing ALT.

Here, we show that abrogation of HR, particularly BRC‐2, in telomerase‐null (*trt‐1*) worms overcomes the early cessation provoked by telomerase deficiency, and this phenomenon is associated with telomere length maintenance. Chromosome fusions were observed, suggesting that telomere uncapping occurred prior to the induction of illegitimate telomere length maintaining mechanism. Furthermore, extra telomeric C‐circles were generated upon depletion of BRC‐2 in *trt‐1* mutants, suggesting that depletion of BRC‐2 can lead to an induction of one form of alternative telomere maintenance. However, BRC‐2 depletion in *trt‐1* mutant worms only partially overcame senescence of *trt‐1* mutants, suggesting that additional mutation may be required for full activation of ALT induction. Taken together, our study suggests that absence of BRC‐2 first results in telomere shortening then induce an illegitimate telomere maintenance mechanism. Our study implies that absence of BRCA2 embraces the possibility to instigate an unidentified ALT pathway in tumorigenesis.

## Materials and methods

### *Caenorhabditis elegans* and feeding RNAi assay

*trt‐1* (*ok410*) worms were grown on RNAi‐expressing lawns of HT115 (DE3) bacteria provided by Dr. Julie Ahringer (Gurdon Institute, Cambridge, UK). Twenty worms at the L3 or L4 stage were passaged every week. Each passage corresponds to two generations. More than 12 sets in each group were tested.

### RT‐PCR in worms

RNAi‐fed worms were lysed and analyzed for knockdown efficiency using RT‐PCR. Primers used for RT‐PCR were as follows: *brc‐2* F, 5′‐TGACAATTGGT‐TCCGATTC‐3′; *brc‐2* R, 5′‐GGATGCTTCTTTTTCGAACG‐3′; *rad‐51* F, 5′‐AAGCTTGCCGATGAATATGG‐3′; *rad‐51* R, 5′‐TTCGGCTTCTGGTAAATTGG‐3′; *rad‐54* F, 5′‐ATGGCGAGGTTTGGAGAGA‐3′; *rad‐54* R, 5′‐TGTGTATCCGATGCCACTGT‐3′; *pot‐1* F, 5′‐GAAAGTTTCCACGCTGCATT‐3′; *pot‐1* R, 5′‐CGCGAACCTTTTCCTGAAT‐3′; *act‐1* F, 5′‐AGGAGTCATGGTCGGTATGG‐3′; *act‐1* R, 5′‐GCTTCAGTGAGGAGGACTGG‐3′.

### Terminal restriction fragment analysis

Genomic DNA was digested with *Mbo*I/*Alu*I (NEB) and separated on a 0.7% agarose gel at 5 V·cm^−1^ at 4 °C and subjected to Southern blotting. After transfer, the DNA was fixed on the membrane by UV cross‐linking, prehybridized with DIG Easy Hyb (Roche, Indianapolis, IN, USA) at 62 °C for 1 h, and then hybridized with telomere‐specific DIG‐labeled probes at 62 °C for 3 h. The blot was washed and the detection was done by exposure to Las‐3000 Imaging System (Fuji Film, Tokyo, Japan) using a DIG Luminescent Detection Kit (Roche).

### Telomere florescent *in situ* hybridization (FISH)

Gonads from 24 h post‐L4 adults were dissected and fixed in 2% paraformaldehyde. Slides were frozen in liquid nitrogen, then immersed in methanol for 10 min at −20 °C, followed by rehydration in 2× SSC containing 0.1% Tween‐20. PNA‐(GCCTAA)_3_ probe was applied in hybridization buffer (2× SSC and 50% formamide). The slides were washed three times with 50% formamide/2× SSC, then counterstained with DAPI and mounted with Vectashield (Vector Laboratories, Burlingame, CA, USA) mounting media. Images were acquired with DeltaVision (Applied Precision, Issaquah, WA, USA), equipped with a 100× objective lens (Olympus, Tokyo, Japan).

### Single telomere length analysis for measuring telomere length

Single telomere length analysis (STELA) in nematodes was performed as described by Cheung and colleagues 8 with some modifications. Briefly, the mixture of telorette oligonucleotide sequences 502–507 was used for ligation: telorette 502, 5′‐GACAGCTATGACTGCTCCGTGCATCTGGCATCGCCTAAG‐3′; telorette 503, 5′‐GACAGCTATGACTGCTCCGTGCATCTGGCATCTAAGCCT‐3′; telorette 504, 5′‐GACAGCTATGACTGCTCCGTGCATCTGGCATCCCTAAGC‐3′; telorette 505, 5′‐GACAGCTATGACTGCTCCGTGCATCTGGCATCCTAAGCC‐3′; telorette 506, 5′‐GACAGCTATGACTGCTCCGTGCATCTGGCATCAAGCCTA‐3′; telorette 507, 5′‐GACAGCTATGACTGCTCCGTGCATCTGGCATCAGCCTAA‐3′.

Amplication was conducted using the following oligonucleotide sequences.

Teltail, 5′‐TGCTCCGTGCATCTGGCATC‐3′

512, 5′‐GATGCGCAGCTAACTATAGGAC‐3′

Worms were lysed for 1 h at 50 °C in lysis buffer (100 mm Tris [pH 8.5], 100 mm NaCl, 50 mm EDTA, 1% SDS, 1% β‐mercaptoethanol, 100 μg·mL^−1^ Proteinase K), and DNA was purified and digested with *Mbo*I/*Alu*I. A mixture of 20 ng DNA and 1 μL of 10 μm telorette primer was incubated for ligation. The ligated DNA was used as the PCR template. PCR products were then separated on a 1% agarose gel. In‐gel hybridization was performed using an end‐labeled telomeric probe.

### Chromosome analysis and counting

Adult hermaphrodites (24 h post‐L4 stage) were soaked in 20 μL of 95% ethanol until evaporate at slide. This reaction was repeated three times. Worms were rehydrated in M9 solution (22 mm KH_2_PO_4_, 42 mm Na_2_HPO_4_, 85 mm NaCl, 1 mm MgSO_4_) and treated with 400 ng·mL^−1^ of DAPI. Slides were mounted with Vectashield (Vector Laboratories). Chromosome numbers of late diakinesis oocyte nuclei were counted under × 1000 magnification, using Axioplan2 microscope (Zeiss, Oberkochen, Germany).

### C‐circle assay

We basically followed the protocol described by Henson *et al*. 9. CC assay was performed with increasing amounts of genomic DNA with same amount of Φ29 DNA polymerase in the reaction. Negative control without Φ29 DNA polymerase was always included. N2 WT control was also employed.

## Results and Discussion

*Caenorhabditis elegans* possesses BRC‐2 (CeBRC‐2), the ortholog of BRCA2, which is only one‐tenth the size of human BRCA2. BRC‐2 in worms is also essential in DNA repair through regulating RAD‐51 filament formation in HR, like its ortholog BRCA2 10. In addition, BRC‐2 in worms also promotes RAD‐51‐independent single‐strand annealing (SSA), replacing Rad52 in mammals 11. Thus, absence of BRC‐2 alone results in low viability in worms.

To assess the physiological outcome of BRCA2 deficiency in telomerase‐deficient situation, we utilized the telomerase‐mutant worm, *trt‐1* and its properties 12. Without telomerase, worms progressively cease to lay eggs around generation 20, accompanied by progressive shortening of telomeres. We hypothesized that by assessing the continuation of generations, one could predict if BRC‐2 depletion synergizes or suppresses further shortening of telomeres. If BRC‐2 depletion synergistically shortens the telomere length, the generation number will be fewer than 20. On the other hand, if depletion of BRC‐2 extends the generations, one could interpret that BRC‐2 may be a suppressor of ALT (alternative lengthening of telomeres). As the generation duration of worms is markedly shorter compared to mammals, this assay is feasible and robust.

**Figure 1:**
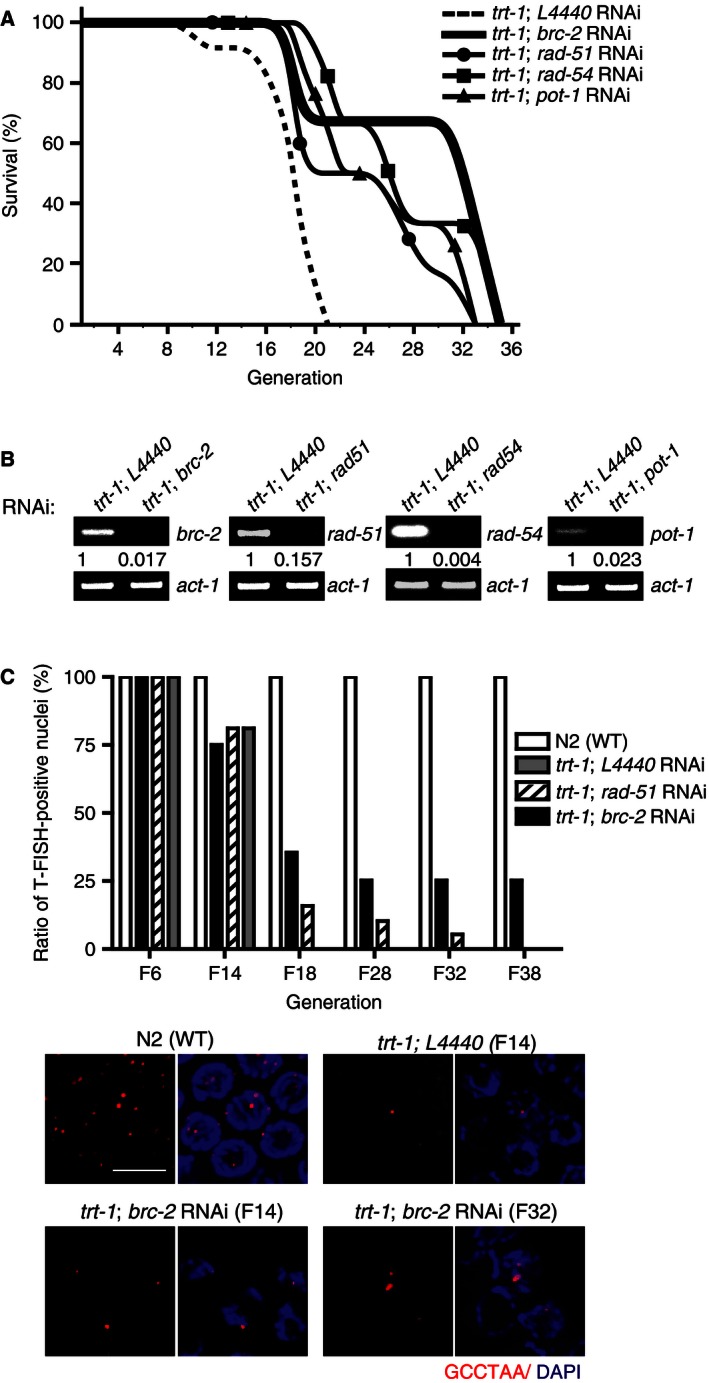
Delay of senescence by the depletion of BRC‐2 in telomerase‐deficient *C. elegans*. (A) Survival rates (%) of telomerase‐deficient *C. elegans* (*trt‐1*) measured in each generation after knockdown of indicated genes. *L4440,* empty vector control. Graph was plotted with the data in Table 1, using B‐spline (OriginPro 8.6). (B) Knockdown efficiency after RNAi in *trt‐1* mutant worms, measured by RT‐PCR, is shown. (C) Ratio of telomere‐containing nuclei in the gonads after telomere‐FISH in different generations (F6 to F38). *X*‐axis, generations; *Y*‐axis, percentage of telomere‐positive nuclei. Representative T‐FISH images from WT *, trt‐1; L4440* (F14), *trt‐1; brc‐2 RNAi* (F14), and *trt‐1; brc‐2 RNAi* (F32) are shown at the bottom. Out of 41 *trt‐1; brc‐2* RNAi cells counted in F32, 27 nuclei exhibited larger telomere foci. Images were taken at same exposure. Telomere was detected by Cy3‐(GCCTAA)_3_ PNA probe (red) and chromatin was counterstained with DAPI (blue). Scale bar, 10 μm.

When BRC‐2 was depleted from *trt‐1* mutants via feeding RNAi method, the generation number was extended to ~ 35 (Fig. 1A and Table 1, *trt‐1*;*brc‐2*), but not beyond, while *trt‐1* mutant worms ceased to lay eggs around generation 21 to 23 (*trt‐1*;* L4440*). The brood size of the worms declined in the initial stage after *brc‐2* depletion (first decline around generation 18), then the generation continued with 67% survival until complete cessation of laying eggs (Table 1). It should be emphasized again that worms with null mutation of *brc‐2* alone are not viable and do not lay eggs because BRC‐2 is critical in meiotic recombination and repair 10. It is interesting that depletion of BRC‐2 in *trt‐1* mutants paradoxically rescues the generations.

**Table 1:** Survival of *trt‐1* mutant worms after RNAi in the indicated generations

	Number of live worms/number of total worms plated (% survival)									
	F1	F12	F18	F19	F21	F22	F27	F29	F33	F35
*trt‐1; L4440*	21/21 (100)	11/12 (92)	9/12 (75)	7/12 (58)	3/12 (25)	0				
*trt‐1; brc‐2*	29/29 (100)	29/29 (100)	10/12 (83)	10/12 (83)	8/12 (67)	8/12 (67)	8/12 (67)	8/12 (67)	4/12 (33)	0
*trt‐1; rad‐51*	25/25 (100)	25/25 (100)	10/12 (83)	6/12 (50)	6/12 (50)	6/12 (50)	4/12 (33)	2/12 (17)	0	
*trt‐1; rad‐54*	25/25 (100)	25/25 (100)	25/25 (100)	25/25 (100)	10/12 (83)	8/12 (67)	4/12 (33)	4/12 (33)	4/12 (33)	0
*trt‐1; pot‐1*	29/29 (100)	29/29 (100)	29/29 (100)	10/12 (83)	8/12 (67)	6/12 (50)	4/12 (33)	4/12 (33)	0	

Telomerase‐deficient *C. elegans* (*trt‐1*) were depleted of the indicated RNA by feeding RNAi method. Surviving worms were counted and replated every week. Initial numbers of worms plated and the surviving worms in indicated generations are shown. Numbers in the bracket refer to the percentage of survival from initial plating.

Depletion of *rad‐51* or *rad‐54*, factors that are essential in HR pathway and in telomere length maintenance 13, was also tested. The levels of knockdown, measured by quantitative RT‐PCR, were similar (Fig. 1B). The result showed that depletion of HR genes also extended the generations (Fig. 1A and Table 1), suggesting that abrogation of HR in the absence of telomerase may have induced an alternative repair pathway. In *C. elegans*, BRC‐2 substitutes Rad52 that is involved in single‐strand annealing (SSA) pathway 11. Therefore, the results collectively suggest that depletion of BRC‐2 in telomerase‐deficient worms instigated a repair pathway that excludes HR and Rad52‐mediated recombination as well. As depletion of BRC‐2 results in progressive shortening of lagging strand telomere synthesis in mammals 7, the paradoxical delay of senescence upon BRC‐2 depletion suggests that dysfunctional BRC‐2 combined with telomerase deficiency leads to a change in telomere environment favorable for illegitimate induction of telomere length maintenance mechanism.

Pot‐1 is essential in telomere capping: it binds to single‐stranded telomeric DNA and protects telomeres from recombination 14. *CeOB2/Pot‐1* depletion in *C. elegans* results in increased heterogeneity in telomere length 15 and abundant single‐stranded C‐rich telomere circles 16, a phenotype similar to that seen in ALT 17. Depletion of POT‐1 in *trt‐1* mutants also extended the generations (Fig. 1A and Table 1), but not completely overcame senescence. This result implies that before the induction of any kind of ALT, telomere uncapping precedes.

To assess whether the delay of senescence was related to the telomere length, we measured the telomere lengths by telomere‐FISH (T‐FISH) in germ cells of various generations. The result was in agreement with the survival studies that there were no telomere‐positive germ cells from *trt‐1* after F18 (Fig. 1C). In comparison, *brc‐2* or *rad‐51* RNAi in *trt‐1* mutants exhibited telomere‐positive nuclei beyond F18: up to F38 in *brc‐2* RNAi worms and F32 in *rad‐51* RNAi‐fed *trt‐1* worms, respectively (Fig. 1C). Interestingly, in F32 of *trt‐1*;* brc‐2* RNAi worms, 65.9% of germ cells (*n* = 41) exhibited larger and brighter T‐FISH signals, even compared to F14 of *trt‐1* or *trt‐1; brc‐2* RNAi worms (Fig. 1C, F32). These bigger T‐FISH spots were not observed in F14. These results suggest that some telomeres were elongated after critical shortening (Fig. 1C, bottom). In the same cell, there were chromosomes with very short telomeres, displaying faint T‐FISH signals as well.

**Figure 2:**
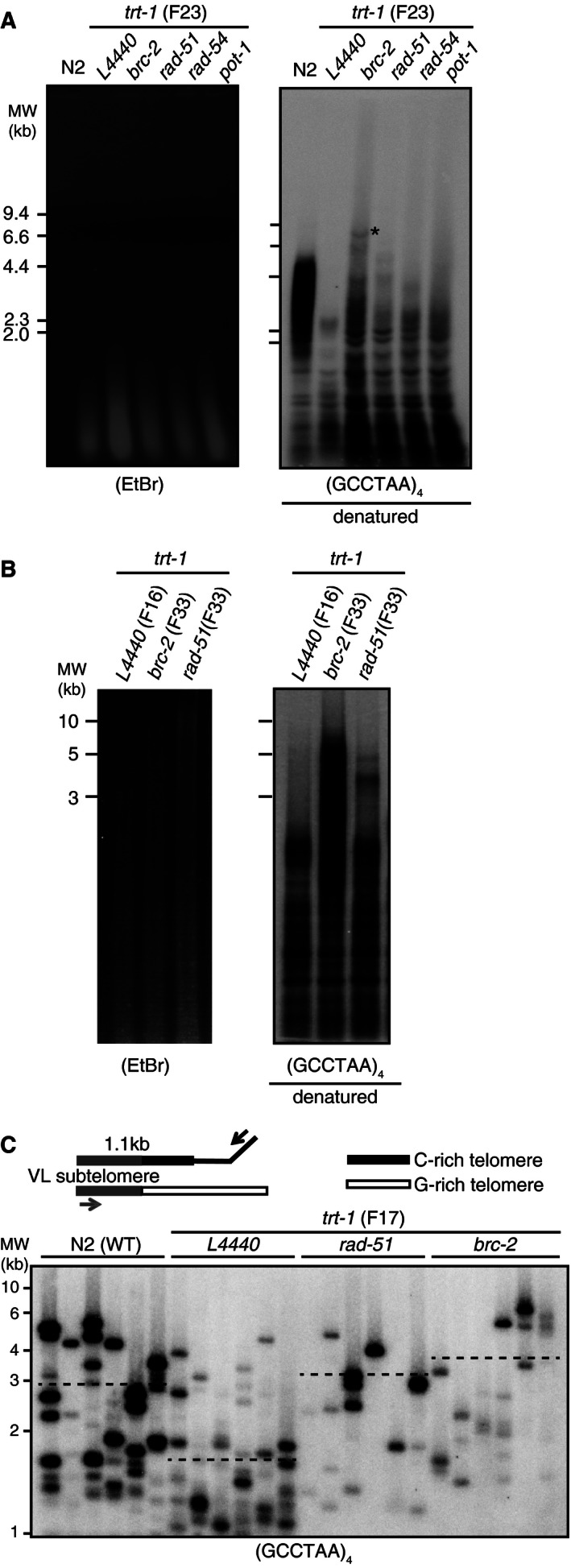
Partial overcome of senescence after the depletion of BRC‐2 in *trt‐1* mutant worms is accompanied by telomere length elongation. (A) TRF analysis of genomic DNA extracted from indicated worms at generation 23 (F23). Capillary transferred digested DNA were hybridized with digoxigenin (DIG)‐labeled telomeric probes and detected with anti‐DIG (right). GCCTAA probe was hybridized. Ethidium bromide‐stained DNA gel before transfer is shown at left and the molecular weight of DNA is marked. Telomeres longer than WT N2 in *trt‐1*;* brc‐2* RNAi is marked with an asterisk. Note that the long smear is not due to degradation but reflects the heterogeneity in telomere length. (B) TRF analysis of *brc‐2* or *rad‐51* RNAi in *trt‐1* mutants in F33, compared to control RNAi in F16. (C) STELA analysis 8 for telomere length measurement. Amplified DNA was separated and detected with an end‐labeled ^32^P‐(GCCTAA)_4_ telomeric probe. Each lane is the result from a single worm. Average telomere length is marked with horizontal broken lines. MW, Molecular weight.

Next, we measured telomere lengths by Southern blot‐based terminal restriction fragment (TRF) analysis. In F23, the telomere lengths of *trt‐1; brc‐2* RNAi worms were more heterogeneous and some were even longer (Fig. 2A, *brc‐2* RNAi, marked with an asterisk), compared to the telomerase‐mutant worms (*trt‐1; L4440*). In F33, telomere lengths of *brc‐2*‐ or *rad‐51*‐depleted *trt‐1* mutants were longer, compared to F16 of *trt‐1* control RNAi worms (Fig. 2B). These data suggested that the telomeres were first shortened, consistent with the result from mouse 7, then were elongated, or at least protected from further shortening, in later generations. Telomere length measurement was further assessed by PCR‐based STELA 8; the average telomere lengths in *trt‐1* worms depleted of *brc‐2* or *rad51* were markedly longer, compared to control *trt‐1* worms (Fig. 2C).

**Figure 3:**
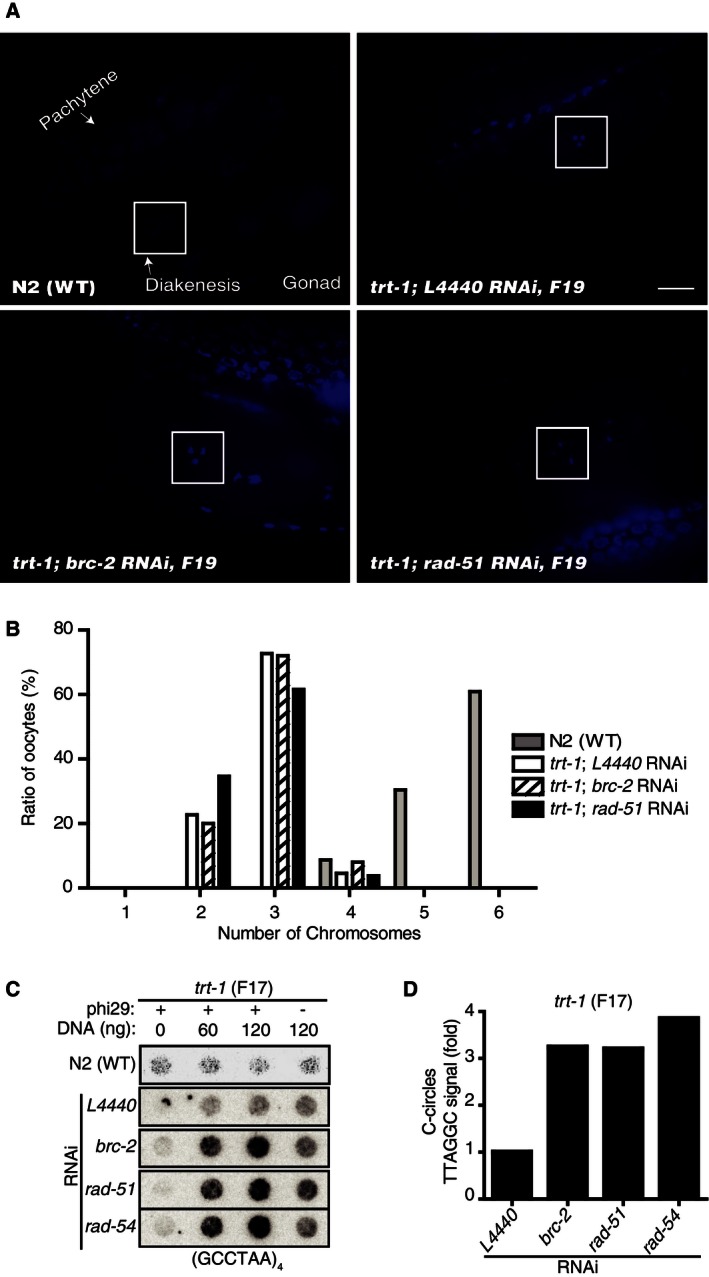
Chromosome structural aberrations in *trt‐1*;* brc‐2* RNAi worms. (A) Representative images of DAPI‐stained late diakinesis nuclei in *trt‐1*;* brc‐2* RNAi oocytes. Pachytene and diakinesis chromosomes in the gonads are marked. Scale bar, 10 μm. (B) Ratio of chromosome fusions, reflected by the chromosome numbers. Chromosomes at generation 19 (F19) of wild‐type N2 (*n* = 23), *trt‐1;L4440* RNAi (*n* = 22), *trt‐1;brc‐2* RNAi (*n* = 25), *trt‐1;rad‐51* RNAi (*n* = 26) were counted and shown in bar graphs. (C) C‐circle assay from F17 *trt‐1* worms after RNAi. Result from N2 is included for control. (D) Bar graph representing the relative level of C‐circles from (C). Signal intensity was normalized to the signal from the background without phi29 (−). Result from N2 WT control is shown. Signal intensity of *trt‐1*;* L4440* RNAi worms was set to 1.

Then we asked whether there was any chromosome alteration in F19, just before the cessation of *trt‐1* mutant worms. As chromosome structures are most visible in germ cells in diakinesis 18, chromosomes in this stage were compared (Fig. 3A). Wild‐type N2 displayed six chromosomes (Fig. 3B, gray bar), whereas *trt‐1* mutants displayed mostly three chromosomes (Fig. 3B, empty bar), suggestive of the end‐end fusions provoked by telomere shortening and erosion 19. In the same generation (F19), depletion of *brc‐2* or *rad‐51* in *trt‐1* mutants also displayed chromosome fusions (Fig. 3B, dashed and black bars), indicating that telomere erosions occurred prior to an induction of alternative telomere repair pathway responsible for the delay of senescence.

As senescence was delayed and telomere shortening was prevented in worms depleted of *brc‐2* in *trt‐1* mutants, we asked whether there was any mark for ALT. Partially single‐stranded telomeric C‐circle (CCCTAA)(n) DNA are the hallmark of ALT in many human cancers 9 and had been detected in worms as well 16. When F17 *trt‐1* mutant worms were subjected to the C‐Circle (CC) assay, approximately fourfold increase of C‐circle abundance was detected in *trt‐1* mutants depleted of the three HR genes (Fig. 3C,D). The result suggests an interesting possibility that an as‐yet‐unidentified ALT can be induced by the depletion of HR in telomerase‐deficient cells. As *trt‐1*;* brc‐2* RNAi worms did not survive beyond F38, a secondary mutation may be required for the full activation of ALT pathway after BRC‐2 depletion. These serial mutations may reflect a path to ALT‐type tumorigenesis. Interestingly, telomere sister chromatid exchange (T‐SCE), an evidence for telomeric recombination, was increased in *BRCA2*‐mutated human cancers 20. How extra telomeric C‐circles are generated after depletion of HR warrants future investigations.

A report from a small number of cell lines showed that depletion of BRCA2 results in two different outcomes: in non‐ALT cells, depletion of BRCA2 leads to the increase of telomere recombination, whereas BRCA2 depletion in ALT cells resulted in the reduction of T‐SCE 21. However, as one ALT cell line used in the analysis, U2OS, cannot represent all ALT pathways, the information cannot be generalized. Here, we are suggesting the path and possibilities of BRC‐2 depletion in inducing illegitimate telomere maintenance pathway, whereas the study from U2OS cell is the assay done at the established cell line. Moreover, U2OS is *BRCA2*‐positive and elongates telomeres via Rad51‐dependent recombination 22. Depleting BRCA2 in U2OS cells is likely to lead to low viability, because its survival depends on Rad51‐mediated telomeric recombination that critically requires BRCA2. Therefore, the report is not in discrepancy with our results. Here, we have showed with compelling lines of evidences that HR deficiency in telomerase‐deficient situation can overcome the senescence. The results shown here suggest that there are many different paths to ALT induction, and that HR deficiency can instigate a novel ALT pathway. As BRCA2 is the tumor suppressor, frequently mutated in human cancers, these results have clinical implications.

To assess the effect of telomere length on senescence, we measured the continuation of generations in worms. In fact, this assay measures the continuance of meiotic recombination, which is distinct from mitosis. Worms depleted of BRC‐2 resulted in a delay of replicative senescence in *trt‐1* mutants, measured by the ability to lay eggs. However, they eventually ceased egg laying (Fig. 1). This result is somewhat different from reported ALT induction where more than 200 generations can be continued in the absence of telomerase 18. BRC‐2 deficiency leads to accumulation of massive DNA adducts on top of telomere erosions, and therefore the failure to propagate beyond F35 may be due to apoptosis. It should be re‐emphasized that worms deficient in *brc‐2* are not viable 10. Hence, adopting additional mutation in genes such as *p53* may fully induce ALT. Indeed, *p53* inactivation frequently accompanies neoplastic transformation of Brca2‐deficient mice 23, consistent with the notion that some familial breast cancers with *BRCA2* mutation in Icelandic population harbor mutation of *p53* as well 24. In summary, BRCA2 suppresses the induction of illegitimate telomere elongation pathway. We do not think that all BRCA2‐deficient cancers exhibit ALT characteristics. Instead, BRCA2‐deficient cells will have very different outcomes depending on whether telomerase is on or off: when telomerase is on, BRCA2‐deficiency will not induce a telomere elongation repair pathway. Taken together, assessing telomere status in BRCA2‐deficient tumors may be helpful in the choice of adequate therapy.

## Author contributions

HL conceived, designed, and coordinated the study. HL also wrote the paper. M‐SK designed and performed key experiments. JM, H‐YJ, KH, and CK performed experiments. JL designed and consulted *C. elegans* experiments. J‐GJ, and W‐YP performed bioinformatical advice. All authors reviewed the results and approved the final version of the manuscript.
